# Link between organic nanovescicles from vegetable kingdom and human cell physiology: intracellular calcium signalling

**DOI:** 10.1186/s12951-024-02340-8

**Published:** 2024-02-19

**Authors:** Martina Trentini, Ilaria Zanolla, Elena Tiengo, Federica Zanotti, Eduardo Sommella, Fabrizio Merciai, Pietro Campiglia, Danilo Licastro, Margherita Degasperi, Luca Lovatti, Massimo Bonora, Alberto Danese, Paolo Pinton, Barbara Zavan

**Affiliations:** 1 Department Translational Medicine, University of Ferrara, 44121 Ferrara, Italy; 2 Departiment of Medical Sciences, University of Ferrara, Ferrara, Italy; 3 Department of Pharmacy, University of Salerno, 84084 Fisciano, SA Italy; 4 AREA Science Park, Padriciano, 99, 34149 Trieste, Italy

**Keywords:** Plant-derived extracellular vesicles, Apple-derived extracellular vesicles, Proteomic, Lipidomic, miRNA, Calcium signalling

## Abstract

**Background:**

Plant-derived nanovesicles (PDNVs) are a novelty in medical and agrifood environments, with several studies exploring their functions and potential applications. Among fruits, apples (sp. *Malus domestica*) have great potential as PDNVs source, given their widespread consumption, substantial waste production, and recognized health benefits. Notably, apple-derived nanovesicles (ADNVs) can interact with human cell lines, triggering anti-inflammatory and antioxidant responses. This work is dedicated to the comprehensive biochemical characterization of apple-derived nanovesicles (ADNVs) through proteomic and lipidomic analysis, and small RNAs sequencing. This research also aims to shed light on the underlying mechanism of action (MOA) when ADNVs interface with human cells, through observation of intracellular calcium signalling in human fibroblasts, and to tackles differences in ADNVs content when isolated from fruits derived from integrated and organic production methods cultivars.

**Results:**

The ADNVs fraction is mainly composed of exocyst-positive organelles (EXPOs) and MVB-derived exosomes, identified through size and molecular markers (Exo70 and TET-3-like proteins). ADNVs’ protein cargo is heterogeneous and exhibits a diverse array of functions, especially in plant's protection (favouring ABA stress-induced signalling, pathogen resistance and Reactive Oxygen Species (ROS) metabolism). Noteworthy plant miRNAs also contribute to phytoprotection. In relation with human cells lines, ADNVs elicit spikes of intracellular Ca^2+^ levels, utilizing the cation as second messenger, and produce an antioxidant effect. Lastly, organic samples yield a substantial increase in ADNV production and are particularly enriched in bioactive lysophospholipids.

**Conclusions:**

We have conclusively demonstrated that ADNVs confer an antioxidant effect upon human cells, through the initiation of a molecular pathway triggered by Ca^2+^ signalling. Within ADNVs, a plethora of bioactive proteins, small RNAs, and lipids have been identified, each possessing well-established functions within the realm of plant biology. While ADNVs predominantly function in plants, to safeguard against pathogenic agents and abiotic stressors, it is noteworthy that proteins with antioxidant power might act as antioxidants within human cells.

**Graphical Abstract:**

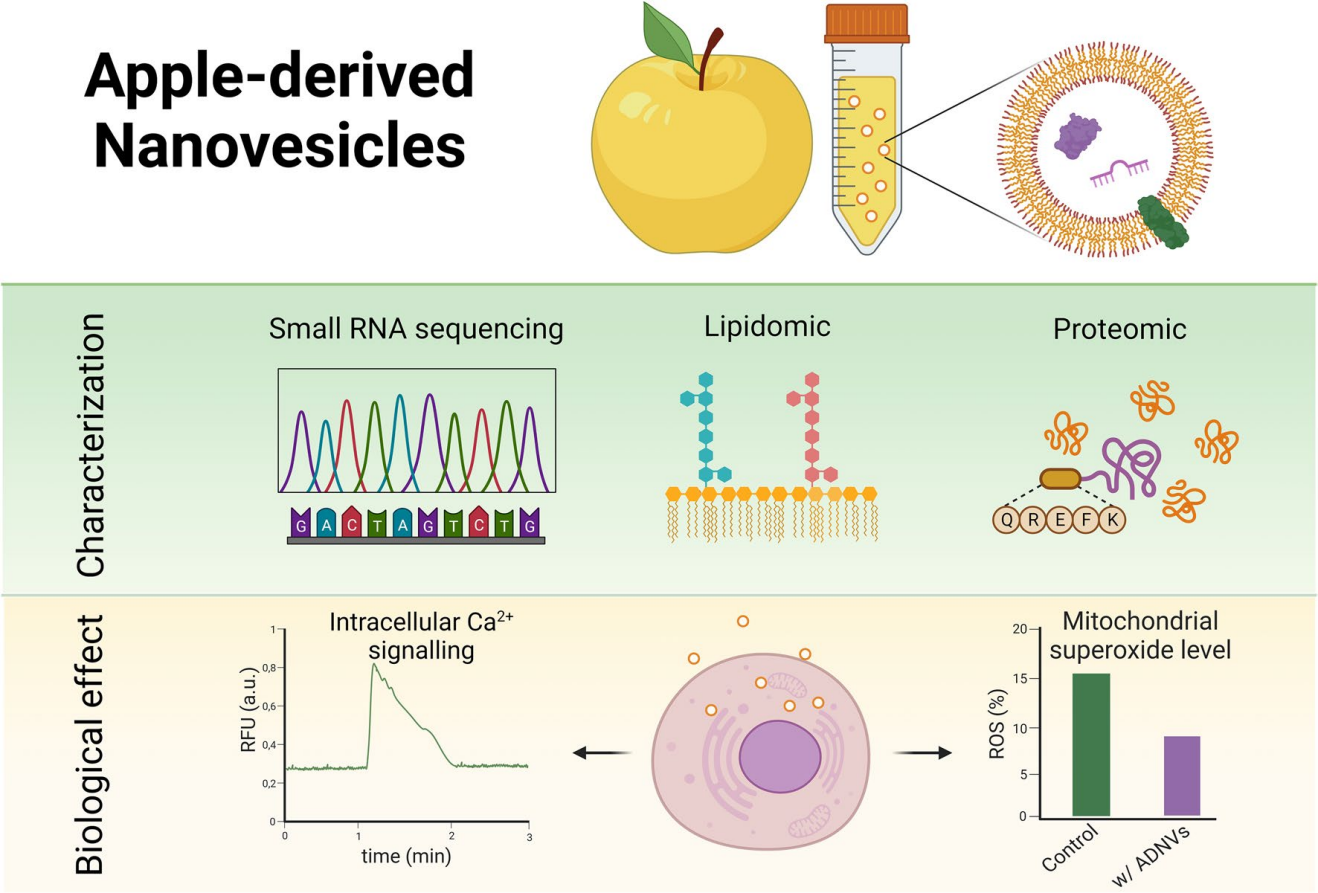

**Supplementary Information:**

The online version contains supplementary material available at 10.1186/s12951-024-02340-8.

## Background

Plant-derived nanovesicles (PDNVs) are double-layered lipidic vesicles. MISEV2018 guideline classifies extracellular vesicles’ population on the base of biogenesis, size, and marker positivity [1, 2]. Plant’s biogenesis of NVs can occur form exocyst-positive organelles (EXPOs), vacuoles, and autophagosomes, while NVs designed as “exosomes” generate by inward budding of multivesicular bodies (MVBs) [3]. From the biogenic and morphogenic points of view, PDNVs are comparable to mammalian-derived EVs [4, 5]. They have a pivotal role in regulating plants' physiological mechanisms via the intercellular transport of bioactive cargos [6], they carry proteins and oligonucleotides mostly involved in plant-pathogen communication through the mutual exchange of signalling molecules, and in the protection against abiotic stress [7–9]. Their content has been proven to be able of cross-kingdom regulation with both mycorrhizal and mammalian species [10, 11].

The therapeutic potential of PDNVs has been a topic of interest in medical research [12–14]. A growing body of evidence suggests that PDNVs have inherent therapeutic properties that benefit human health [15–17]. PDNVs are also being explored as natural options for targeted drug delivery systems [3, 18, 19]. Their employment could help overcome issues with drug bioavailability and off-target effects [3, 20, 21]. Indeed, PDNVs are biocompatible and remain stable in the human body even when administered orally [22–25].

Apple (*Malus domestica*) is a well-known fruit of economic and ecologic relevance. According to the “Prognosfruit 2022” report, EU countries have harvested 12.2 million metric tons of fresh apples crop in 2022. It has already been established that nanovesicles are contained in *Malus domestica*’s fruit [26–29]. In a prior investigation involving nanovesicles sourced from apples (referred to as ADNVs), we established their capacity to influence cellular processes and exhibit antioxidant potential within human cells [28]. This inquiry revealed the phenomenon of ADNVs interacting with human macrophages (derived from the THP-1 cell line). These interactions induced a remarkable effect: a transition of the macrophage phenotype from the pro-inflammatory M1 phenotype to the regenerative-promoting M2 phenotype, thereby attenuating inflammation and fostering a regenerative environment. A separate preceding study unveiled that the anti-inflammatory influence of ADNVs stretches its effects across diverse cell types, encompassing dermal fibroblasts. Here, the subdued inflammation response was correlated with a decline in Reactive Oxygen Species (ROS) levels and a simultaneous increase in collagen synthesis. Evidently, ADNVs have demonstrated a constructive impact on tissue regeneration and the revitalization of skin, indicating their potential as viable active constituents for formulations within the realm of cosmetics [29]. However, the mechanisms promoting such biological effects are unknown.

Organic certified production avoids utilizing certain pesticides and chemicals during the plant life cycle (Regulation (EU) 2018/848), thus modifying the soil microbiome. By its nature, organic farming produces higher volumes of residual fruit, not suitable for the market. With the intent of exploiting ADNVs for health-related approaches, the fruit source is a crucial economic and ecological choice that remains to be made.

The purpose of the present work is to characterize the composition of ADNVs in terms of their protein, lipid, nucleotide cargo and to define the basal mode of action activated as soon after the treatment of the cells with them. We aim to understand the mechanisms behind ADNVs' cross-kingdom interaction and enhance our knowledge of PDNVs and their potential uses. Additionally, given the potential salutary properties of PDNVs, understanding if organic farming practices impact their composition could have important implications for ADNVs' source choice for future applications.

## Materials and methods

### EVs isolation from whole fruit

Apple-derived NVs (ADNVs) were isolated from DOP Golden variety apple cultivar fruit (*Malus domestica* sp.), cultivated in Val Di Non (Trentino, Italy). Apples were selected from two distinct ‘Golden Delicious’ diploid cultivar conditions, organic (GDBio) and integrated (GD). The isolation protocol is described by [29]. Briefly, four apples of approximately 250 g each were washed thoroughly. Juice was extracted through cold press mechanism. The released pulp, comprised of fibrous and liquid fractions, was subjected to a series of three centrifugations at increasing speed (650 rcf, 3.000 rcf and 10.000 rcf), to gradually remove denser and denser debris from the solution. At the end of each centrifugation step, the EVs-enriched supernatant was transferred in a novel tube, while the fibrous fraction was discharged. After centrifugation at 10.000 rcf, the EVs-enriched fraction was filtered at 0,22 µm with vacuum filtering systems for 10 min (Sartorius, Germany). The solution was centrifuged at 15.000 rcf with Ultracentrifuge Optima L-70 (Beckman Coulter Inc., USA), type 70 Ti rotor, to remove macrovesicles and cell-wall debris. The supernatant was further centrifuged at 110.000 rcf, to deposit low-density EVs. The pellet, resulting from this last centrifugation, was resuspended in 1 mL of PBS (Thermo Fisher Scientific, MA, USA), and was used as ADNVs-enriched fraction in all following experiments. All ADNVs-enriched fractions were conserved at – 80 °C until use.

### ADNVs quantification and size characterisation

Following isolation, ADNVs were quantified through Tuneable Resistive Pulse Sensing (TRPS) (qNANO Gold, Izon Science Ltd, Cambridge, MA, USA). The analysis also provided data on size distribution of particles in the ADNVs-enriched fraction. The nanopore (NP100, Izon Science Ltd, Cambridge, MA, USA) was stretched 49 mm wide and measured with a digital calliper. Pore installation, wetting and cleaning was performed with reagents provided by the manufacturer, following manufacturer’s instructions. As standard procedure, each sample was measured both at 10 atm and 20 atm of pressure. During each measurement, particle rate was maintained above 200 particles/minutes and the total particle count surpassed 500 particles. Calibration particles (CPC100, Izon Science Ltd, Cambridge, MA, USA) were used for calibrating each sample measurement during data analysis, at 10 atm and 20 atm, ensuring a maximum current divergence of 5%. Each measurement was performed in triplicate.

### Cell culture

Fibroblasts from the derma were cultured in Dulbecco’s Modified Medium (DMEM) completed with 10% Foetal Bovine Serum. For both Ca^2+^ signalling analysis and mitochondrial superoxide assay, cells were seeded onto coverslips or plates respectively, and cultured for 24 h before the beginning of the experiment. For the Mitochondrial Superoxide Assay and ABTS Assay, fibroblasts were treated with TNFα (100 ng/mL) for 6 h. Cells were then treated with 0, 100, 200 and 300 μg of ADNVs (GD), dissolved in 1 mL of DMEM complete medium for 12 h.

### *Ca*^*2*+^*signalling analysis*

The cytosolic Ca2 + response was evaluated using the fluorescent Ca^2+^ indicator Fura-2 (Life Technologies, Invitrogen). Fibroblasts, grown onto 24-mm coverlids, were incubated for 30 min at 37 °C in 1 mM Ca2 + /KRB supplemented with 2,5 mM Fura-2 AM, 0.02% Pluronic F-68 (SigmaAldrich), and 0.1 mM sulfinpyrazone (Sigma-Aldrich). For samples necessitating extracellular Ca^2+^ removal, the above-descripted medium was spiked with EGTA (5 μM). At the end of incubation, coverslips were washed and supplemented with either 1 mM Ca2 + /KRB or 1 mM Ca2 + /KRB + 5 μM EGTA. The live cells were placed in an open Leyden chamber on a 37 °C thermostat-controlled stage. Acquisition was performed by exposing the cells to 340 nm/380 nm wavelength light, using the Olympus xCellence multiple-wavelength fluorescence microscopy system, equipped with an ORCA ER CCD camera (Hamamatsu Photonics) and an Uplan FLN 40 × oil objective (Olympus). The data, expressed as emission ratio, was then plotted as a curve, and the area underneath (AUC) was calculated to compare conditions.

### Mitochondrial superoxide assay

After treatment, cells were assayed with MitoSOX™ Mitochondrial Superoxide Indicators (Thermo Fisher Scientific), as for manufacturer’s instructions. Cells were then detached from the culture plate and resuspended in 25 μL of PBS. The MitoSOX dye permeates cellular membrane of live cells, is oxidized by superoxide, and produces red fluorescence. Fluorescence was measured with Tali Image-Based Cytometer (Thermo Fisher Scientific) at absorption/emission 396/610 nm. Un-stained cells were used for background reference.

### DPHH assay

Radical scavenging assay was performed with the radical DPPH (1,1-diphenyl-2-picrylhydrazyl), as described by Yu et al. [30]. A solution of 400 μg/mL DPPH in methanol was prepared, filtered with syringe filters at 0,22 μm and kept away from light. To test ADNVs scavenger ability, ADNVs at different concentrations (100, 200 and 300 μg/mL) were added to the DPPH solution at 1:1 ratio. The solutions were mixed and incubated at RT for 30 min. Samples were placed in a 96 well, two wells per-sample, and absorbance was measured at 516 nm with a plate reader (Victor3, PerkinElmer, MA, USA). Results are shown as percentage relative to the control sample (DPPH), after normalization over the blank sample (methanol). The experiment was performed in triplicate.

### TEM imaging of ADNVs

Both GD and GDBio ADNVs fractions were prepared for Transmission Electron Microscopy (TEM), imaging following a procedure for the staining of extracellular vesicles described by Corona et al., 2023 [31]. Briefly, EVs were deposited at a concentration of 10^9^ particles/mL and a TEM Grids 200 Mesh Cu/Pd. Sample and grid were fixed with 2% paraformaldehyde (PFA) 1% glutaraldehyde in 100 mM PBS, pH 7,4 solution. For staining, a contrasting solution of Methylcellulose/Uranyl Acetate was applied for 10 min. The grid was dried, and pictures of the grid were taken with TEM Zeiss EM 910 instrument (Carl Zeiss Microscopy, Oberkochen, German). Images were recorded by a CCD digital camera (Ultrascan 1000, Gatan, Munich, Germany).

### Proteomics sample preparation and nLC-MS/MS analysis

Total protein content was assessed though Bradford assay with Pierce™ BCA Protein Assay kit (Thermo Fisher Scientific, Waltham, MA, USA) following manufacturer’s instructions. Absorbance was measured at 570 nm using multilabel plate reader Victor 3 (Perkin Elmer, Milano, Italy). ADNVs of both GD and GDBio conditions were digested with trypsin as previously reported [32]: After digestion and clean-up, MS analysis was performed by nanoflow ultra-high performance liquid chromatography-high resolution mass spectrometry using an Ultimate 3.000 nanoLC (Thermo Fisher Scientific, Bremen, Germany) coupled to an Orbitrap Lumos tribrid mass spectrometer (Thermo Fisher Scientific) with an Easy nano electrospray ion source (Thermo Fisher Scientific). Peptides were trapped in a PepMap trap column (Thermo Fisher), and then loaded and separated onto a C18-reversed phase column (250 mm × 75 μm I.D, 2.6 µm, 100Å, Biozen). Mobile phases were A): 0,1% HCOOH in water v/v; B): 0,1% HCOOH in ACN/Water v/v 80/20, a linear 60 min gradient was performed. HRMS analysis was performed in data dependent acquisition (DDA), with MS1 range 375–1.500 m/z, HCD fragmentation was used with normalized collision energy setting 27. Resolution was set at 120.000 for MS1 and 15.000 for MS/MS. Single and unassigned charge were excluded. Quadrupole isolation was set to 3Da. Each sample was analysed by LC–MS/MS in duplicate. MS data were acquired using a data-dependent method, dynamically choosing the most abundant precursor ions from the survey scan (375–1.500 m/z) using HCD fragmentation. Maximum ion injection times for MS (OT) and the MS/MS (OT) scans were set to auto and 50 ms respectively, and ACG values were set to standard. Dynamic exclusion: 30 s. For data processing, raw MS data were analysed using Proteome Discoverer v 2.5 (Thermo Fisher) MS/MS was matched against Malus Domestica proteome (Uniprot 03/2022 version). The following parameters were used: enzyme trypsin, missed cleavages max 1, mass accuracy tolerance 10 ppm and 0,6 Da for precursors and fragments respectively. Sequest search and Percolator algorithm were used. Carbamidomethylcysteine was used as fixed modification while methionine oxidation as variable. Proteins were considered identified with at least one unique peptide, using a false discovery rate (FDR) threshold of < 0,1. Each analysis was performed in triplicate.

### Lipid sample preparation and UHPLC-TIMS analysis

Lipid extraction and mass spectrometry‐based lipid detection were performed on ADNVs derived from GD and GDBio samples, in triplicate. 225 µL of cold MeOH (methanol) containing a mix of deuterated standards (EquiSPLASH® LIPIDOMIX®, Avanti Polar Lipids, Alabaster, AL, U.S.A) were added to ADNVs, vortexed and then incubated for 1 min at -30°C. The samples were shacked and sonicated for 10 min (× 2). Subsequently, 800 µL of cold MTBE (methyl tert-butyl ether) were transferred to the tube and the solution was incubated in a thermomixer (Eppendorf, Hamburg, Germany) for 1 h, 500 rpm at 4 °C. To induce phase separation, 188 µL of H_2_O were added and samples were centrifuged at 14680 rpm, for 10 min at 4 °C. The upper layer was collected and evaporated using a SpeedVac (Savant, Thermo Scientific, Milan, Italy). The dried samples were dissolved in 100 µL of CHCl_3_/MeOH/IPA 1/2/4 (*v/v %*) before the UHPLC-TIMS analysis. Unless otherwise described, all solvents and additives were LC–MS grade and purchased by Merck (Darmstadt, Germany).

UHPLC-TIMS analyses were performed on a Thermo Ultimate RS 3000 coupled online to a TIMS-TOF-Pro quadrupole Time of flight (Q-TOF) (Bruker Daltonics, Bremen, Germany) equipped with an Apollo II electrospray ionization (ESI) probe, as previously reported [32]. The separation was performed with an Acquity UPLC CSH C18 column (100 × 2,1 mm; 1,7 μm) protected with a VanGuard CSH precolumn (5,0 × 2,1 mm; 1,7 μm, 130 Å) (Waters, Milford, MA, U.S.A). The column temperature was set at 55 °C, a flow rate of 0,4 mL/min was used, mobile phase consisted of (A) ACN/H_2_O containing HCOONH_4_ (10 mM) and 0,1% HCOOH 60:40 (*v/v %*) and (B) IPA/ACN containing HCOONH_4_ (10 mM) and 0,1% HCOOH 90:10 (*v/v %*). The following gradient has been used: 0 min, 40% B; 2 min, 43% B; 2.10 min, 50% B; 12 min, 54% B; 12.10 min, 80% B; 15 min, 99% B; 17 min, 99% B, 17.10 min, 40% B and then 2.9 min for column re-equilibration. The analyses were performed in data-dependent parallel accumulation serial fragmentation (DDA-PASEF) with both positive and negative ionization, in separate runs. The oven temperature was set to 55°C and the injection volume was 2 µL for the positive mode and 5 µL for the negative mode.

Source parameters: Nebulizer gas (N_2_) pressure: 3,0 Bar, Dry gas (N_2_): 10 L/min, Dry temperature: 250°C. Mass spectra were recorded in the range m/z 50–1500, with an accumulation and ramp time to 100 ms each. The ion mobility was scanned from 0,55 to 1,80 Vs/cm^2^. Precursors for data-dependent acquisition were isolated within ± 2 m/z and fragmented with an ion mobility-dependent collision energy ranging from to 20–40 eV in positive mode while a fixed collision energy (40 eV) was employed in negative mode. The total acquisition cycle was of 0.32 s and comprised one full TIMS-MS scan and two PASEF MS/MS scans. Exclusion time was set to 0.1 min, Ion charge control (ICC) was set to 7.5 Mio. The instrument was calibrated for both mass and mobility using the ESI-L Low Concentration Tuning Mix with the following composition: [m/z, 1/K_0_: (322,0; 0,7 Vs cm^−2^), (622,0; 1,0 Vs cm^−2^), (922,0; 1,2 Vs cm^−2^), (1222,0; 1,4 Vs cm^−2^)] in positive mode and [m/z, 1/K_0_: (302,0; 0,7 Vs cm^−2^), (602,0; 0,9 Vs cm^−2^), (1034,0; 1,2 Vs cm^−2^), (1334,0; 1,4 Vs cm^−2^)] in negative mode.

4D data alignment, filtering and annotation was performed with MetaboScape 2021 (Bruker) employing a feature finding algorithm (T-Rex 4D) that automatically extracts buckets from raw files. Feature detection was set to 500 and 250 counts for positive and negative modes. The minimum number of data points in the 4D TIMS space was set to 100, and recursive feature extraction was used. The spectra were processed in positive mode using [M + H]^+^, [M + Na]^+^, [M + K]^+^, [M + H-H_2_O]^+^ and [M + NH_4_]^+^ ions in positive mode, while [M–H]^−^, [M + Cl]^−^, [M + HCOO]^−^ and [M + H-H_2_O]^+^ in negative mode, the assignment of the molecular formula was performed for the detected features using Smart Formula™ (SF). Lipid annotation was performed first with a rule-based annotation and subsequently using the LipidBlast spectral library of MS DIAL (http://prime.psc.riken.jp/compms/msdial/main.html) with the following parameters: tolerance: narrow 2 ppm, wide 10 ppm; mSigma: narrow 30, wide 250, MS/MS score: narrow 800, wide 150. CCS%: narrow 2, wide 3. For the assessment of repeatability and instrument stability over time was used a mixture of lipid standards [LightSPLASH®, Avanti Polar Lipids], blank samples were used to assess and exclude background signals. At the end all lipids missing more than 75% of samples or influenced by carry-over effects were deleted. Lipids were quantified using the corresponding deuterated internal standard as reported previously [33]. Each analysis was performed in triplicate.

### Small RNA sequencing

Small RNAs were isolated from ADNVs samples of GD and GDBio derivation with Exosome Purification and Exosomal RNA Isolation kit (Norgen Biotek Corp., Thorold, ON, Canada) following manufacturer instructions. The extracted RNA quality and concentration was verified with NanoDrop One (Thermo Fisher Scientific, Waltham, MA, USA). RNA was then stored at – 80 °C until use.

Sequencing of all miRNAs was performed with Illumina sequencing technology. Briefly, 250ng of RNAs were processed using QIAseq miRNA Library Kit (QIAGEN; Hilden, GE). Sequencing was performed on a Novaseq 6000 Sequencing System (Illumina; San Diego, CA, USA) in 2 × 150 paired-end mode. Identification of miRNAs in the samples was performed using the QIAseq miRNA-NGS data analysis software considering Single Read as read type and Read 1 Cycles 75 as read cycles. Each analysis was performed in triplicate.

### Enrichment and statistical analysis

Pathway enrichment analysis was performed on proteomics results, following “Pathway enrichment analysis and visualization of omics data using g:Profiler, GSEA, Cytoscape and EnrichmentMap” nature protocol [34]. Briefly, normalized output from GD and GDBio proteomics were enriched using g:Profiler open-access online enrichment software (https://biit.cs.ut.ee/gprofiler/gost). Parameters were chosen as follows: *Malus domestica* organism, significance was calculated by the software with FDR-adjusted p-value (p-value_adj_) < 0,05, and multiple testing correction was performed with g:GOSt tailor-made algorithm g:SCS for reducing significance score. Enrichment was performed on three different data sources, biological process (BP), cellular compartment (CC) and molecular functions (MF) on gene set database Gene Ontology (GO). “Inferred from electronic annotations” (IEA) matches were not removed, since the specie *Malus domestica* is less-well-studied compared to the human genome and model organisms. For the same reason, gene set size was not reduced during following steps of enrichment analysis. Therefore, larger and smaller pathways are included. Enrichment data was uploaded on network visualization software Cytoscape, where EnrichmentMap application was used to create the networks of pathways. A hierarchical layout was chosen for the visualization, with operator manipulation. For data interpretation, AutoAnnotate application was used to cluster pathways into groups with a summarized name, to provide an overview of the enrichment result themes.

miRNA sequences were aligned with *Malus domestica* transcriptome though Qiagen analysis software Ingenuity Pathway Analysis (IPA). Screening of miRNAs was performed by selecting miRNA present in three or more sample replicates with an average mean READ count abundance over 100. Abundance figures display the READs mean of all replicates with ± standard error (SE), they were obtained with GraphPad Prism 8 software version 8.0.0 for Windows (GraphPad Software, San Diego, California USA, www.graphpad.com). Comparison between GD and GDBio samples was analysed statistically by one-way ANOVA, followed by the Bonferroni post-hoc multiple comparison test (GraphPad Software). Differences between groups were considered significant given p-values < 0,05.

Lipid profiling was conducted with LipidSig (http://chenglab.cmu.edu.tw/lipidsig/). Univariate statistics and pathway analysis was performed with open-access software MetaboAnalyst 5.0 (https://www.metaboanalyst.ca/). Pathway analysis was performed by selecting *Arabidopsis thaliana* KEGG pathway library, using all compounds of the pathway library, though Hypergeometric test type enrichment. A scatter plot was produced for the visualization of significant features.

Principal Component Analysis (PCA) plots, Volcano plots and Heatmaps of significant proteins, miRNAs or lipids were produced with SRplot (https://www.bioinformatics.com.cn/en) online tool (cluster orientation: bidirectional; cluster method: complete; distance method: Euclidean).

## Results

### ADNVs characterization

**Fig. 1:**
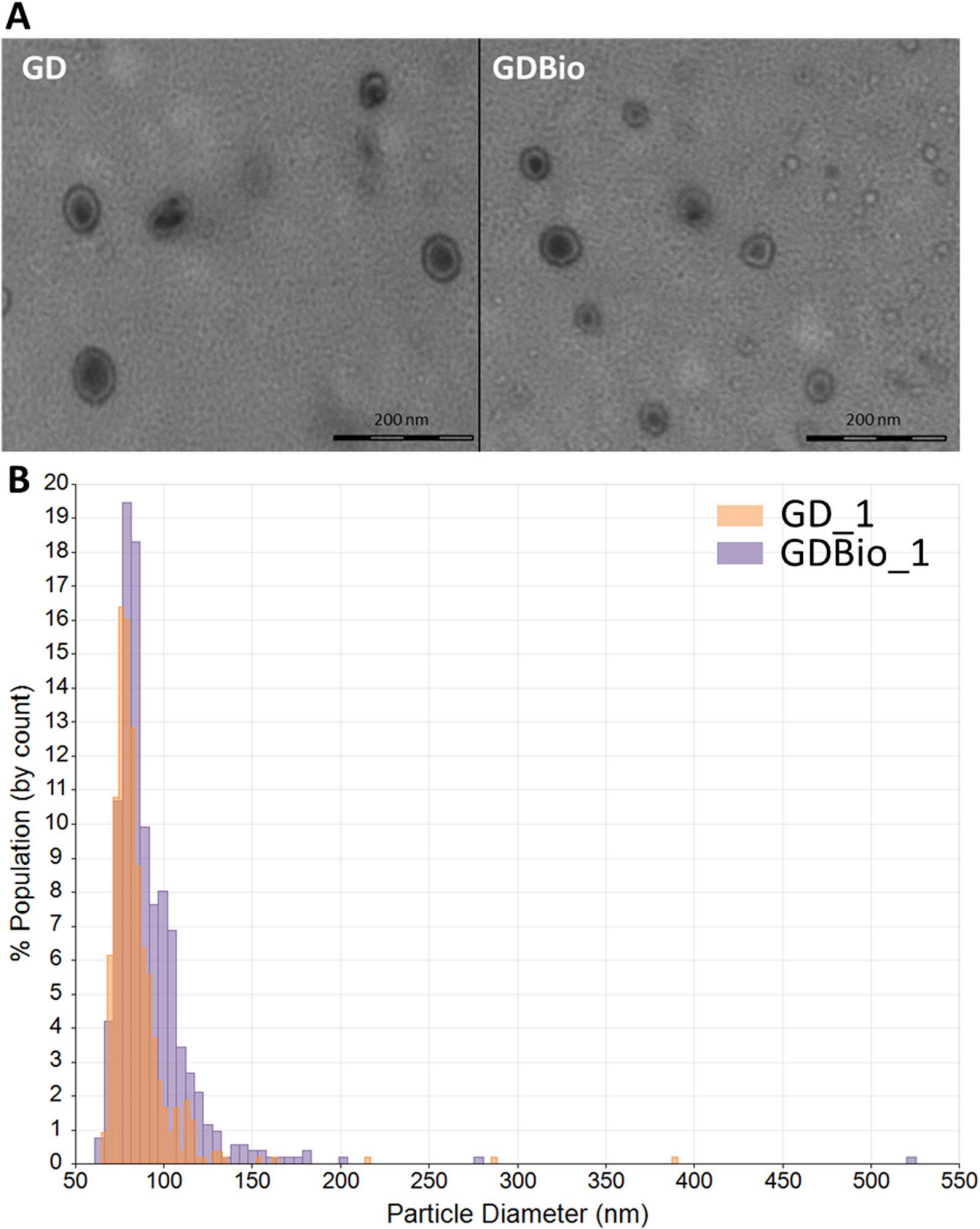
Quantification, size distribution and morphological characterization of ADNVs. **A** TEM Imaging of ADNVs from GD and GDBio samples; **B** TRPS analysis output. The graph represents the distribution size (diameter) in nm, correlated to the population percentage for each bean

We sought to quantify and physically characterize ADNVs isolated from ‘Golden Delicious’ apple fruits, both from integrated (GD) and organic (GDBio) production method and to determine whether cultivation practices influence their abundance or structure. The TRPS quantification and size distribution analysis outcome is shown in Fig. 1B, while details on each run are shared in Table 1. Organic farming practice does not seem to influence the size distribution of ADNVs, as the particle’s size ranges from 50 to 150 nm, with few outliers up to 450 nm in both GD and GDBio samples. The most densely populated range is between 50 and 85 nm, with an average mode diameter ranging from 68 to 74 nm. However, raw particle concentration differs in the two conditions. Integrated production (GD) yields 2,6E + 09 particles/mL, while GDBio yields 1,5E + 11 particles/mL. Lastly, ADNVs morphology was examined through Transmission Electron Microscopy (TEM). In Fig. 1A, ADNVs are visible as round-shaped objects, ranging from 60 to 300 nm, in accordance with TRPS dimension analysis.

**Table 1:** TRPS analysis data

Sample	Mean Diameter (nm)	Mode Diameter (nm)	Raw Conc (/mL)	Particle Count	Particle Rate (/mL)
GD_1	85,0	76,0	2,3E + 09	537,0	712,0
GD_2	82,0	77,0	4,7E + 09	515,0	1254,0
GD_3	86,0	78,0	3,2E + 09	524,0	783,0
GDBio_1	92,0	79,0	2,7E + 11	551,0	1381,0
GDBio_2	89,0	80,0	2,5E + 11	556,0	1315,6
GDBio_3	89,0	78,0	2,2E + 11	682,0	1153,7

### ADNVs protein characterization

The protein cargo of ADNVs, isolated from GD samples, was identified through LC–MS/MS-based quantitative proteomic analysis. In total, 187 proteins belonging to *Malus Domestica* were identified with high confidence in ADNVs of GD samples. Additional file 2: Table S1 shows the complete list of proteins, their UniProt entry code and their full name. *Malus domestica* proteome has not been extensively studied yet. For this reason, 37,5% of proteins in this dataset are tagged as uncharacterized, 30% are recognized based on one or more domains and only 32% are known proteins (Additional file 1: Figure S1).

**Fig. 2:**
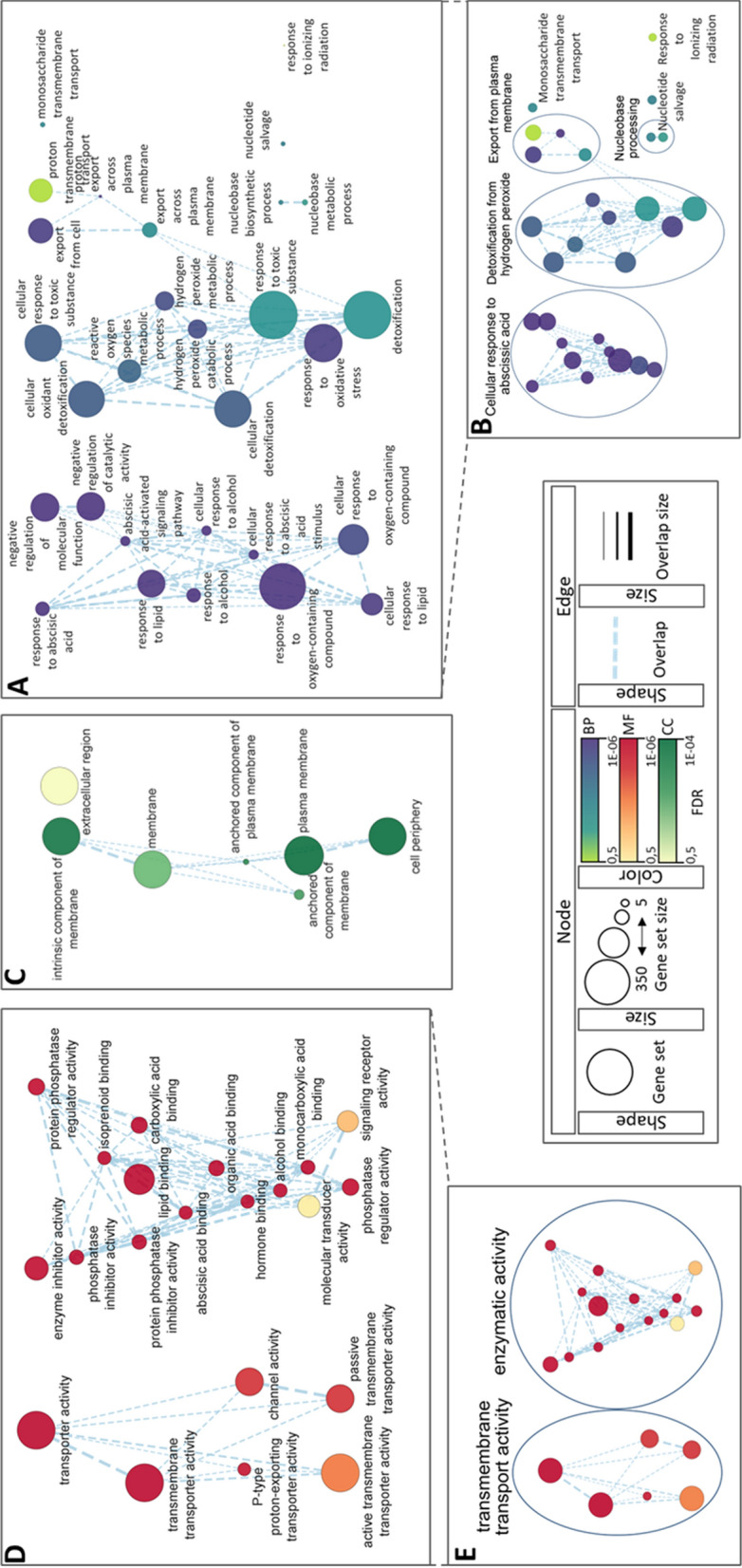
Networks obtained from Cystoscape visualization software, following enrichment analysis of proteomic outcome with g: Profiler online software. **A, B** The figure shows Biological Process (BP) network of all proteins (**A**), each node is labeled with the pathway entry name (in reference to GO database), and (**B**) their clustering through AutoAnnotate tool; **C** Cellular Compartment (CC) network of said proteins as for BP; **D–E** Molecular Function (MF) pathway analysis showing all pathways (**D**) and pathway clustering with annotation (**E**) as for BP

A follow-up pathway analysis of the proteomic results was performed to determine protein involvement in biological pathways (BP), molecular functions (MF) and cellular compartment (CC) (Fig. 2, Additional file 2: Table S2). BP analysis is shown in Fig. 2A, where twenty-six pathways have been selected for visualization. Through the AutoAnnotate application (Fig. 2B), we were able to identify three main pathway clusters. The first cluster is related to abscisic acid (ABA) isoprenoid phytohormone response and is the cluster with lower p-value_adj_ node values, thus the most significant. Notable nodes belonging to this cluster are “response to oxygen-containing compounds” (GO:190170) and "abscisic acid-activated signalling pathway" (GO: 0009738). The latter is a child term to "cellular response to oxygen-containing compound" (GO:1901701), "response to lipid" (GO:0033993), "response to alcohol" (GO:0097305), "response to hormone" (GO:0009725), "response to abscisic acid" (GO:0009737) and "cellular response to abscisic acid stimulus" (GO:0071215). All these terms are contained in the first cluster. The second cluster is related to the oxidative stress response, more specifically to the detoxification from hydrogen peroxide. Most genes in this cluster are grouped under the general GO entries “detoxification” (GO:0098754), “response to oxidative stress” (GO:0006979) and "response to toxic substance" (GO:0009636). "Hydrogen peroxide catabolic process" (GO:0042744) is a child term for "reactive oxygen species metabolic process" (GO:0072593) and for "hydrogen peroxide metabolic process" (GO:0042743), both also present in this cluster. The third and smallest cluster pertains to the export from plasma membrane. Among these nodes, the most statistically relevant are “export from cell” (GO:0140352) and “proton export across plasma membrane” (GO:0120029). Lastly, four unrelated nodes have been omitted from the clustering (Fig. 2B), while a smaller cluster comprised by two nodes is tagged “nucleobase processing”.

The three main aspects of BP, identified through auto-annotation, are mirrored in the MF and CC classification of said proteins (Fig. 2C, D and E). The MF enrichments classified genes into 21 functions, clustered into two: transmembrane transport activity and enzymatic activity. "Transporter activity on the peripheral membrane" (GO:0022857) and, in more general terms, "transporter activity" (GO:0005215) are the two most populated and most significant nodes. Other functions, classified under enzymatic activities, are also bound to the extracellular membrane: “lipid binding” (GO:0008289), “ABA binding” (GO:0010427), and “phosphatase inhibition” (GO:0019212) (Fig. 2E). Accordingly, CC enrichment placed most proteins in the "cell periphery" compartment (GO:0071944) (Fig. 2C). More specifically, several proteins have been classified as "intrinsic components of membrane" with few "anchored components of plasma membrane", although these terms are obsolete. Quite a few proteins are placed under the “extracellular region” (GO:0005576) term, but with lower significance.

### Organic farming influence on ADNVs protein cargo

**Fig. 3:**
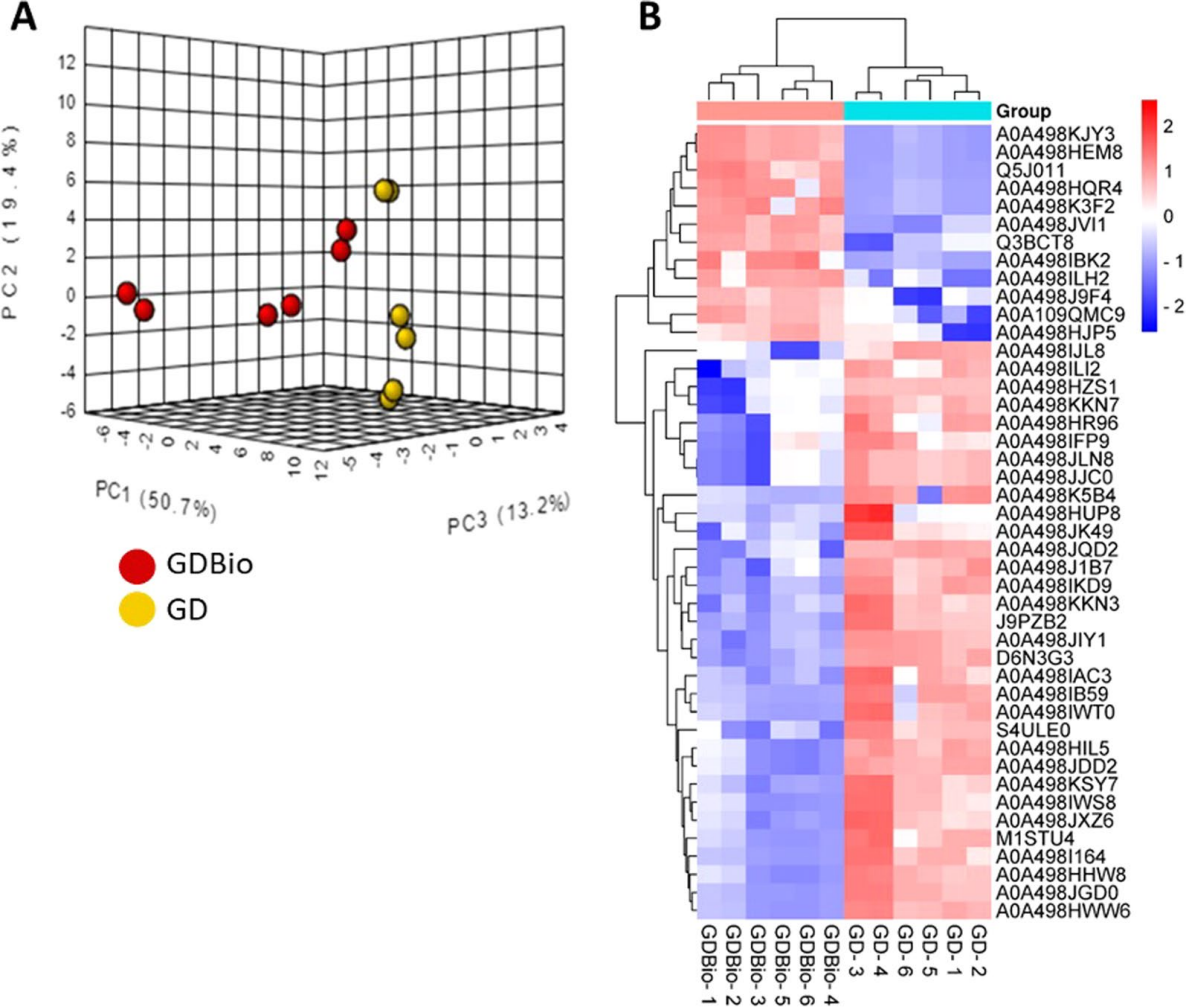
Comparison of proteomic results between GD and GDBio samples. **A** Principal Component Analysis (PCA) of GD (yellow) and GDBio (red) samples pertaining the protein characterization of samples; **B** Significant proteins’ expression in GD and GDBio samples replicate

A comparison of protein cargo has been made between ADNVs isolated from GD and GDBio production methods. PCA plot illustrates a summary of the information contained in each GD and GDBio replicate. In the illustration, we can observe divergent tendencies among the biological replicates of GD and GDBio (Fig. 3A). However, the heatmap of Fig. 3B of the proteomic profile revealed clustering among replicates and differences between conditions. Of 64 proteins, that have been detected in both GD and GDBio (Additional file 2: Table S4), 44 are differentially expressed following the t-test cut-off (Fig. 3B). Hence, the proteomic analysis confirmed that alternative cultivation methods altered ADNVs’ protein cargo. Pathway analysis was unavailable for differentially expressed genes since the number of known proteins is too scarce (Additional file 1: Figure S1). Nonetheless, investigation of the significant results showed peroxidases (A0A498KKN7, A0A498JLN8), dehydrin 9 (J9PZB2), plasma membrane ATPase (A0A498I164) and VAMP (M1STU4) are overexpressed in GD sample, while non-specific lipid-transfer protein (Q5J011), superoxide dismutase (A0A498K3F2) and thaumatin-like protein (Q3BCT8) are overexpressed in GDBio vesicles.

### Characterization of the small RNA population in ADNVs

**Fig. 4:**
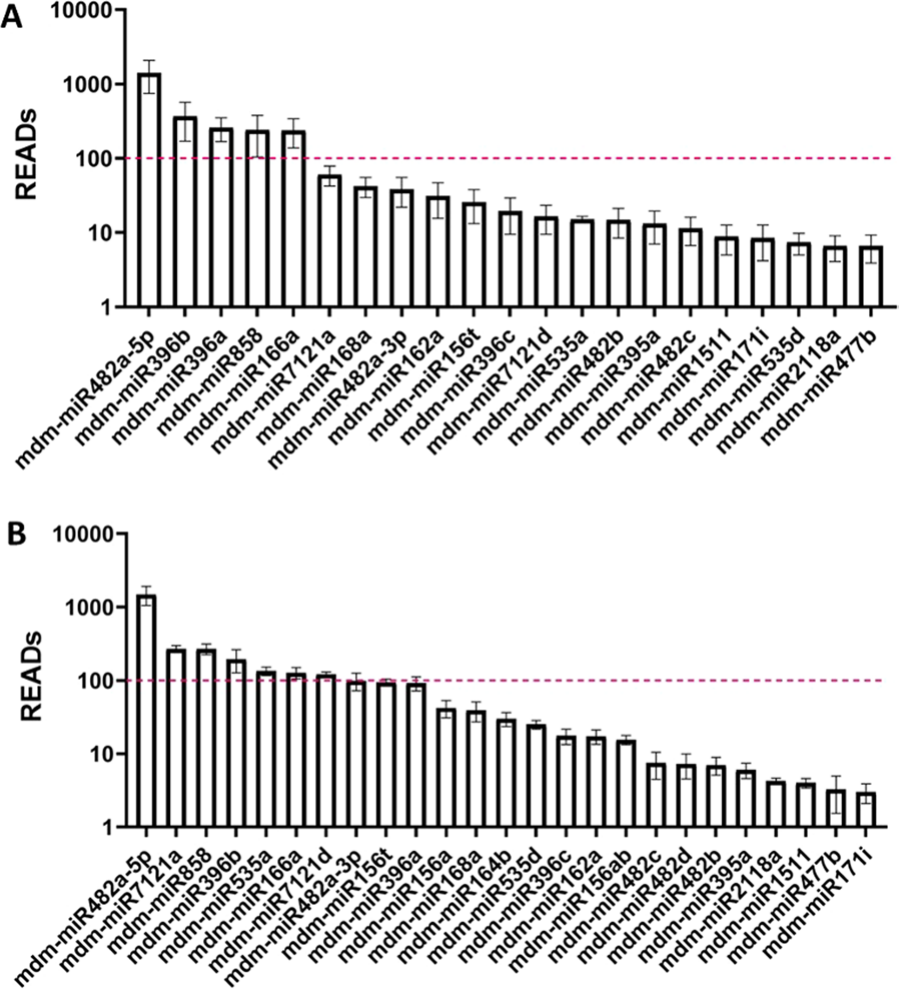
miRNA expression in GD (**A**) and GDBio (**B**) samples. The graph reports READs value on a logarithmic (log_10_) scale. The dotted line represents READs value of 100

Small RNA sequencing was employed to investigate the nucleic acid content of ADNVs, in both GD and GDBio conditions (Additional file 2: Table S3). By confronting the obtained sequences with miRbase database, *Malus domestica* organism, we revealed 20 miRNA families in GD exosomes (Fig. 4A). The most abundant miRNAs, with READ counts above the established cut-off, are mdm- miR482a-5p, mdm-miR396b, mdm-miR396a, mdm-miR858 and mdm-miR166a family representatives.

Although the nucleic acid content in GD and GDBio is similar in nature, the latter partially deviates from the first. Twenty-five miRNAs were identified in GDBio (Fig. 4B), of which mdm-miR482a-5p, mdm-miR7121a, mdm-miR858, mdm-miR396, mdm-miR535, mdm-miR166 and mdm-miR7121 reached the threshold. To better understand the influence of organic farming practices on ADNVs cargo, we need a visual comparison of the two datasets. Additional file 1: Figure S2A shows fold change (FC) and statistical significance (-log_10_(p-value)) of miRNAs in GDBio compared to GD. Nine are over-represented in GDBio, organic farming sample (mdm-miR156ab, mdm-miR164b, mdm-miR535a, mdm-miR535d, mdm-miR7121a, mdm-miR7121d) while eight in GD farming samples (mdm-miR396a, mdm-miR166a/b/c/d/e/f/g/h/i, mdm-miR1511, mdm-miR162a/b, mdm-miR482c, mdm-miR482b, mdm-miR171i, mdm-miR396b). However, after filtering again for a minimum average count READs > 100, miRNAs mdm-miR164, mdm-miR1511 and mdm-miR162 were excluded. The complete list of miRNAs, their FC in GDBio vs GD and -log_10_(p-value) is shown in Additional file 1: Figure S2B.

### Lipidomic analysis and enrichment

**Fig. 5:**
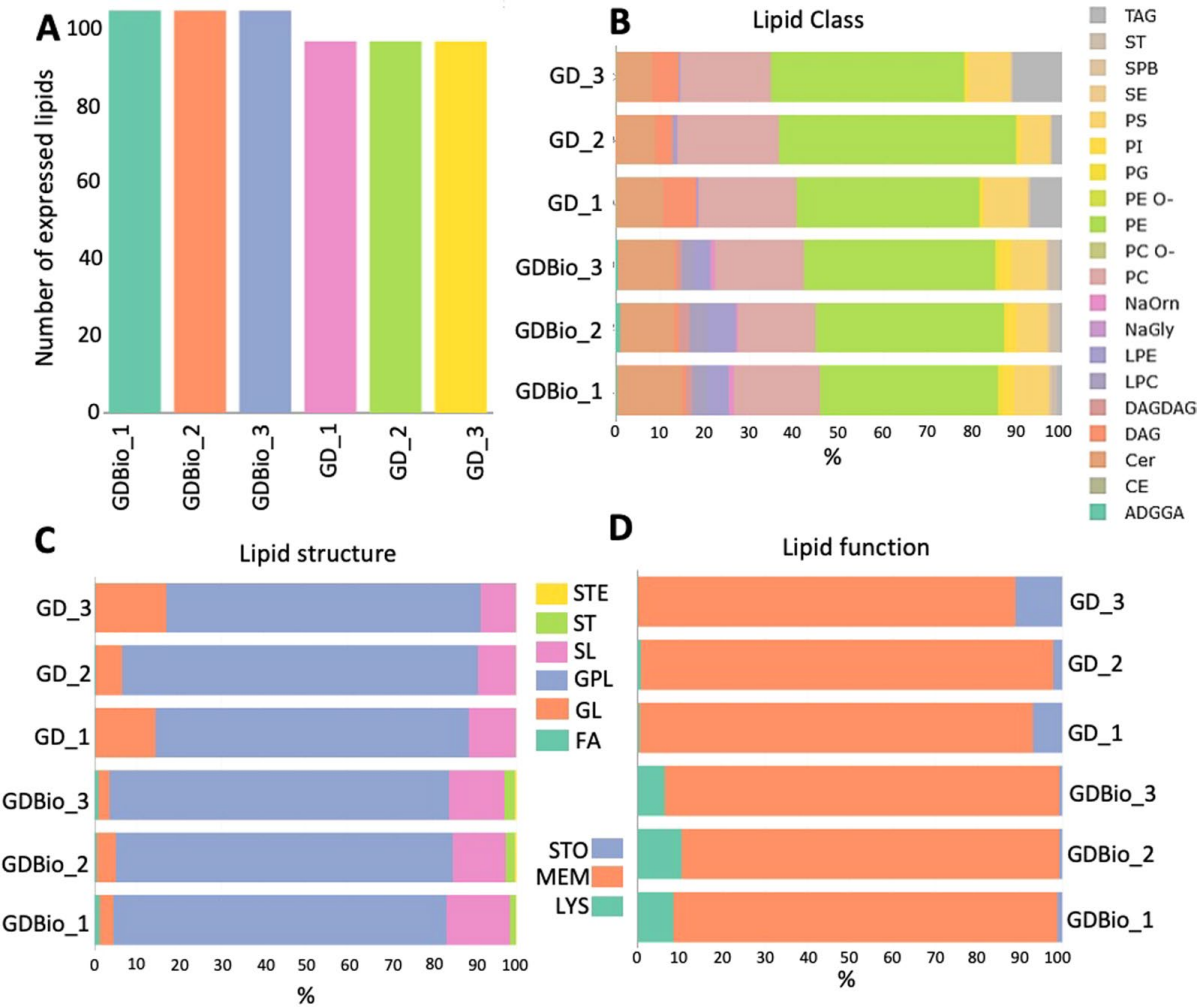
**A–D** Lipid classification for each sample belonging to the GD and GDBio clusters. **A** Overall lipid content in each sample replica; **B** Lipidic function distribution in membrane (MEM), storage compartment (STO) and lysosome (LYS); **C** Characterization of lipidic structure in sterol esters (STE), sterols (ST), sphingolipids (SL), glycophospholipids (GPL), glycolipids (GL) and fatty acids (FA); **D** Lipidic class distribution as percentage

Lipids in EVs represent the outer double-layered enveloping coating. Understanding the lipid composition of EVs is one starting point for understanding the interaction mechanism of EVs with recipient cells. Therefore, lipidomic analysis was performed on both GD and GDBio conditions. As shown in Fig. 5A, lipid species abundance is comparable among samples of the same and different derivations. From this analysis, derived 158 putative unique lipids annotations (a complete list is reported in Additional file 2: Table S4). Functional analysis of said lipids revealed that in all samples, more than 90% (c.a.) of lipids belong to the plasma membrane (MEM) compartment (Fig. 5D). The remaining 10% (c.a.) hails from lysosomes (LYS) in GDBio samples, and from storage compartments (STO) in GD samples.

Lipids can be further classified by their structure (Fig. 5C). In both GD and GDBio samples, at least 80% (c.a.) of entries are glycerophospholipids (GPL), with a slight difference in percentages of glycolipids (GL) and sphingolipids (SL). 19 different subclasses were identified, Fig. 5B represents the composition percentage of lipids in each sample. Circa 40 to 60% of lipids are phosphatidylethanolamines followed, in both conditions, by phosphatidylcholines (PC), ceramides (Cer), and phosphatidylserine (PS). The heatmap in Additional file 1: Figure S3 highlights statistically significant differences in lipid classes among samples by hierarchical clustering. GD samples exhibit a higher proportion of diacylglycerols (DAG), triacylglycerols (TAG), Cer, PC, and phosphatidylethanolamine (PE) compared to GDBio, while the latter is richer in lysophosphatidylcholines (LPC), lysophosphatidylethanolamines (LPE) and phosphatidylinositol (PI). As for the single lipid species, GD and GDBio diverge in composition (Additional file 1: Figure S4 and S5). According to volcano plot analysis, 11 lipidic species differ significantly (Additional file 1: Figure S5). GD sample is two times more enriched in PC 20:0_18:2_A, but poor of LPE 18:2, LPC 18:2_A and LPC 18:1 compared to GDBio.

**Fig. 6:**
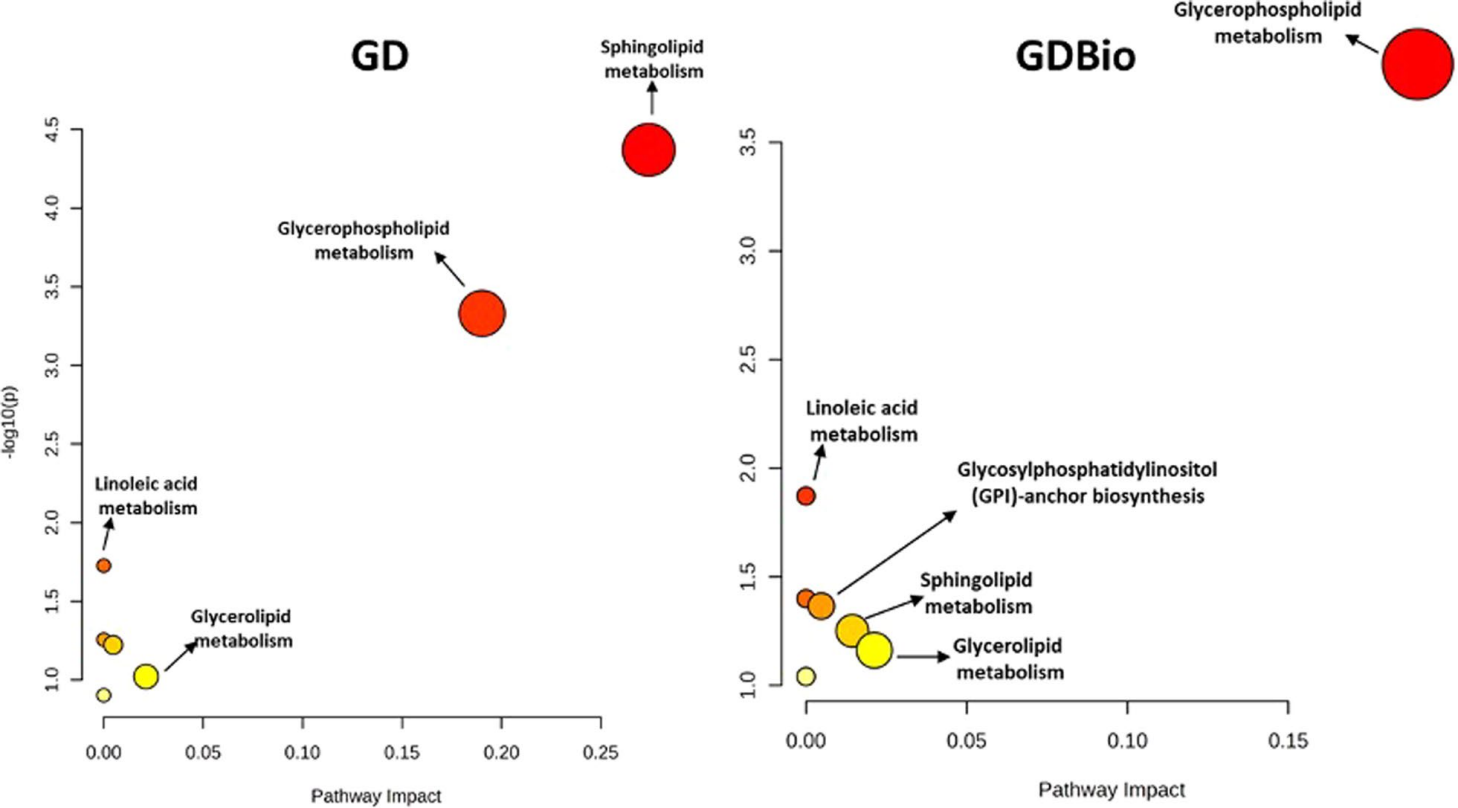
Pathway analysis on lipidomic profiles, performed with MetaboAnalyst 5.0. for GD and GDBio samples. “Pathway impact” represents a combination of the centrality and pathway enrichment results; higher impact values represent the relative importance of the pathway; circle size indicates the impact of the pathway, the colour (from yellow to red) represents the significance which is also represented by –log_10_(p-value)

Given lipidic importance in exosome interaction with the environment, pathway enrichment was performed for GD ADNVs. Pathway analysis highlighted seven enriched pathways, shown in Fig. 6. GDBio lipids are less involved in sphingolipid metabolism and more in linoleic acid metabolism, with little difference if compared to GD samples. The enrichment ratio measured as the number of genes linking the pathways and each lipid category, points out that lipids whose functions had been most enriched mostly belong to the GPL class (glycerophosphocholines, glycerol phosphoethanolamines and glycerol phosphoserines) and to sphingoid bases (Additional file 1: Figure S6).

### *Ca*^*2*+^*signalling*

**Fig. 7:**
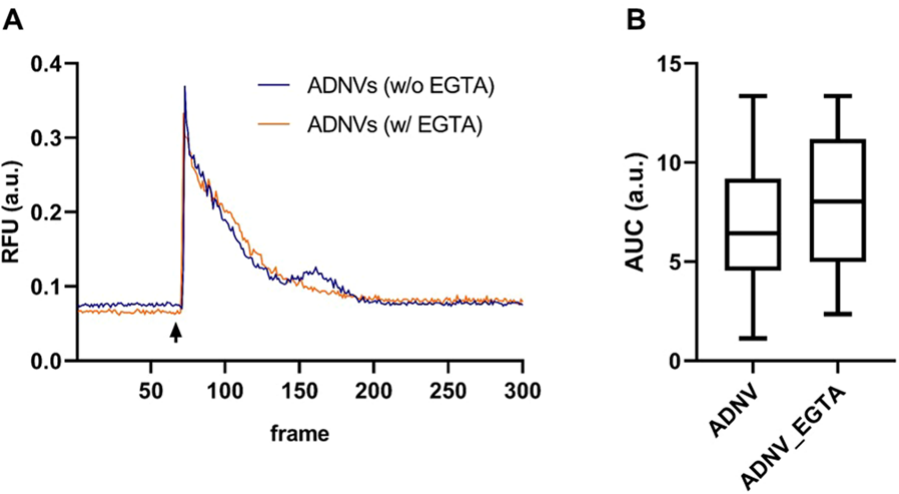
Intracellular Ca^2+^ levels. **A** The curves are representations of intracellular calcium variations, depicted as a ratio of the Relative Fluorescence Units (RFU) and frames, in cells standing in a medium with or without EGTA. The arrow indicates the addition of ADNVs to the cell medium; **B** Area Under the Curve (AUC) values for calcium variation provoked by ADNVs addition, in cells standing in a medium with or without EGTA. There is no statistical significance between the two distributions

The calcium ion is a ubiquitous intracellular messenger, that controls diverse cellular functions. Various cell stimuli, including extracellular signalling molecules such as ADNVs, can cause alterations in Ca^2+^ homeostatic mechanisms. The increase of cytosolic Ca^2+^ concentration results from either (i) the influx of extracellular Ca^2+^ via the plasma membrane Ca^2+^ channels, or (ii) the release of Ca^2+^ from internal stores. Our results show that ADNVs promote a sudden increase in cytosolic Ca^2+^ concentration, that subsides with time, returning to the initially established baseline concentration (Fig. 7A). We also determine that the addition of Ca^2+^ chelating agent (EGTA) in the extracellular compartment still produces a Ca^2+^ peak after ADNVs stimulation (Fig. 7A). By confronting the Area Under each Curve (AUC), we have observed that Ca^2+^ intensity peaks in conditions of presence or absence of EGTA, are equal in mean and distribution (Fig. 7B). Therefore, the above-mentioned spike is not caused by influx of extracellular Ca^2+^.

### Antioxidant activity

**Fig. 8:**
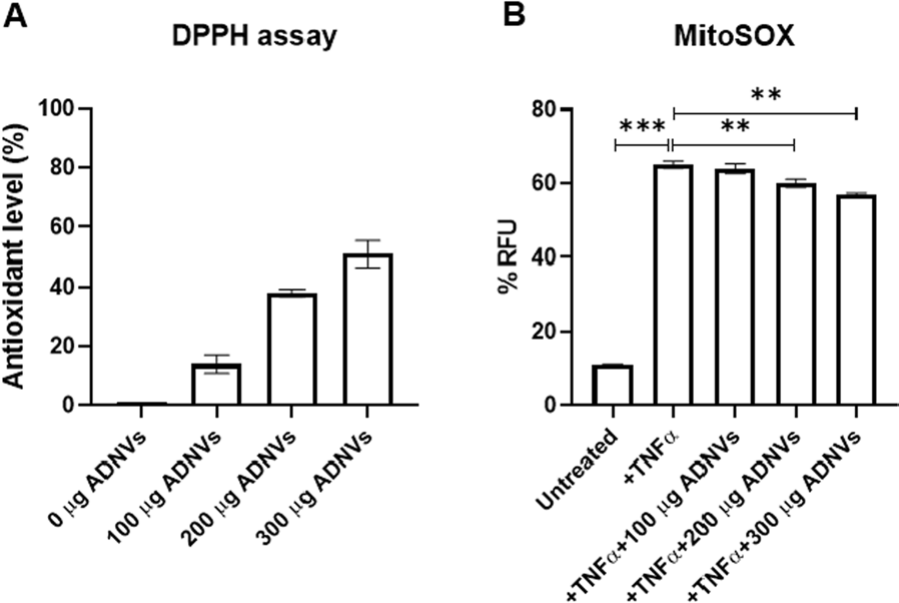
**A** Total antioxidant capacity. Measure of the ABTS + scavenging ratio of ADNVs, expressed as percentage relative to the control condition. **B** Mitochondrial superoxide level. The Relative Fluorescence Unit (RFU), normalized on the number of cells in each sample, is compared between cells treated with TNFα, cells treated with TNFα and ADNVs at increasing concentrations, and untreated cells. *p < 0.05, ** p < 0.01, *** p < 0.001 and **** p < *0.0001*

The antioxidant potential was measured with colorimetric DPPH assay. Results show that the DPPH is reduced by ADNVs to DPPHH (Fig. 8A). The scavenging activity of ADNV increases with its concentration. To further investigate the role of ADNVs in scavenging cellular Reactive Oxygen Species (ROS), we have increased superoxide production through treatment with TNFα, which can stimulate the production of superoxide though the activation of NADPH oxidases [35]. Mitochondria are considered as the main source of reactive oxygen species (ROS) in the cell, and the superoxide anion an undesirable by-product of mitochondrial oxidative phosphorylation. Therefore, mitochondrial superoxides were quantified in treated and untreated cells. As shown in Fig. 8B, the addition of ADNVs leads to significant reduction in superoxides in live cells, at both treatments 200 μg and 300 μg ADNVs. Treatment with 100 μg ADNVs did not produce significant changes in mitochondrial superoxide levels.

## Discussion

### ADNVs markers for EVs subpopulations prediction

When isolating EVs from a complex vegetal matrix, like the one presented in this article, the identification of EV subpopulations in the purified fraction is essential for understanding the subsequent molecular and biological characterization. Here, we attempt to create a parallelism between how EVs have been previously characterized in plants and NVs from *Malus domestica*. Size-based characterization of GD samples shows that ADNVs range from 50 to 150 nm (Fig. 1B), and can be therefore be referred to as “small EVs population” [2]. Proteomic analysis has revealed the presence of the Exo70 family protein, involved in EXPOs mediate exocytosis [36] and of tetraspanin-3-like protein (A0A498IQK8) (Additional file 2: Table S1). TET-3-like presents tetraspanin-8-related domains, including four transmembrane helices (all predicted). Plant-derived exosomes (PDExos) are enriched in proteins of the tetraspanin superfamily (TET). Particularly, TET-8 has been evaluated as PDExos marker in several occasions [8, 37]. The detection of Exo70 and TET-3-like proves that the ADNVs-enriched fraction, of both GD and GDBio, is composed by vesicles of heterogeneous origin. We can identify two main groups: EXPOs and MVB-derived exosomes. Nonetheless, the mere protein detection in this proteomic assay cannot allow TET-3-like to be regarded as an apple-derived exosome marker without previous in vitro validation. Moreover, the lipidomic profile of ADNVs (GD) showed a large quantity of PE, PC and PS, all of which have roles as structural lipids and in the regulation of PM-protein interactions (Fig. 5B). Other studies have reported that PDEVs are enriched in PC and PE, compared to their cell-of-origin’s PM [38]. PS and PI (Fig. 5B) are detected in most endomembrane compartments including MVBs [39]. The few PI species present in ADNVs samples might be involved in the “GPI-anchor biosynthesis pathway” (Fig. 6). The lipidomic profile of ADNVs is therefore consistent with their predicted nature and biogenesis, and similar to other PDEVs.

### Biological effect on human fibroblasts

As mentioned in previous works, ADNVs can interact with human cells in in vitro conditions. ADNVs induce changes in the polarization of macrophages from a pro-inflammatory type (M1) to a non-inflammatory type (M2) [28]. Moreover, they have an anti-oxidant, anti-inflammatory effect of human fibroblasts [29]. We have indeed confirmed that ADNVs reduce superoxide species produced in the mitochondria of stressed cells (Fig. 8B).

In this study, we have gone deeper into the understanding of ADNVs Mode Of Action (MOA), to determine the above-mentioned effects. Cytosolic calcium variation is commonly observed, as calcium is a second messenger, mediator of several cellular function. ATP-driven pumps and specific channels maintains the cation’s homeostasis, for the cytosolic concentration needs to be kept c.a. 100 times lower than its concentration in the extracellular compartment (EC). The EC and intracellular organelles sequester Ca^2+^ and, in response to appropriate signals such as extracellular stimulus, release it back into the cytoplasm. The Ca^2+^ release pattern is finely tuned, to be able to trigger different functions in the same cell [40]. When cellular Ca^2+^ concentration is in overload and the machine put in place to maintain homeostasis is debilitated, it produces a highly toxic response resolving in cell death [30]. The cytosolic Ca^2+^ spike, observed after 2–5 s after ADNVs injection in cell media, is indication that (i) ADNVs biological effect is mediated by Ca^2+^ signalling, and (ii) that the signal, returning then to the baseline cytosolic Ca^2+^ concentration after the spike, does not lead to cell death. Interestingly, in absence of extracellular Ca^2+^ ADNVs still produce equal effect of Ca^2+^ variation. Therefore, we can conclude that the Ca^2+^ wave induced by ADNVs interaction with the cell does not come from the EC, but from one internal compartment. Reservoirs of Ca^2+^ are the endoplasmic reticulum ER and the Golgi apparatus, in crosstalk with the mitochondria. Further studies need to establish which of these intracellular compartments is responsible for the Ca^2+^ wave induced by ADNVs.

### ADNVs’ protective role in plant

Several studies have shown that PDNVs loaded cargo is involved in various defence mechanisms [41–43]. The present work suggests that ADNVs are carriers of similar content. Proteins involved in plant-pathogen interaction are abundant in ADNVs isolated from GD samples (Fig. 2). As pathway analysis indicates, ADNVs are enriched in proteins involved with ABA signalling (Fig. 2A, B). ABA is a phytohormone that regulates a wide variety of physiological processes in plants, including the response to biotic or environmental stress. How ADNVs are implicated in ABA signalling can be understood by observing ADNVs’ cargo protein families. Firstly, we identified numerous isoforms of PM H( +)-ATPase. The presence of H( +)-ATPase 4 in tomato NVs was proven an effective attack against plant pathogens by apoplastic acidification [44, 45]. Lipid-transfer proteins (LTPs) found in ADNVs are also involved in pathogen resistance [46–49]. A model proposed by Madni et al. [50] suggests that LTPs might bleach lipids on fungal membranes, thus increasing their porosity [50]. Yet another group of proteins found in ADNVs are peroxidases, enzymes that belong to the antioxidant machinery, put in place by plants against abiotic or biotic stress, also found in tomato-derived EVs [51, 52]. Lastly, literature brings plenty of evidence for the role of the exocyst proteins, such as Exo70 found in ADNVs, in innate plant immunity [53–57]. The presence of the aforementioned proteins ultimately suggests an active part of ADNVs in aggression against intruding pathogens, by protoplast acidification and membrane bleaching, and indirectly by reducing damage by reactive species.

Small RNAs also play a significant role in conferring stress resistance across both the animal and plant kingdoms, through post-transcriptional regulatory mechanisms. Among apple plants' miRNAs, mdm-miR858 is one of the most characterized, for its negative regulation of pro-anthocyanidins biosynthesis [58]. Anthocyanins, important secondary metabolites with antioxidant activities, are deployed for contrasting oxidative stress and are contribute to the fruit redness. miRNA mdm-miR156 family is likewise implicated in the anthocyanin accumulation process, contributing to the homeostasis of antioxidants [59–61]. Strikingly, both of these miRNAs have been identified within ADNVs (Fig. 4A). Additionally, another noteworthy miRNA family carried by ADNVs is mdm-miR482, which targets mRNA coding for the plant invertase superfamily (group of enzymes with a role in defence response against fungal pathogens) [62], and is involved in tobacco mosaic virus resistance [59].

Lastly, ADNVs are enriched in lipids involved in linoleic acid metabolism (Figs. 6). Linoleic acid reduces oxidative stress by promoting the repair of proteins damaged by oxidation [63, 64].

In conclusion, we can formulate a hypothesis suggesting that ADNVs are active part of the plant’s immune system. They engage in direct defence mechanisms through apoplastic acidification and membrane bleaching and orchestrate a comprehensive systemic response through the transfer of miRNAs between cells. Additionally, akin to other PDNVs, these entities might serve as concentrated reservoirs of oxidation-reducing compounds. This proposition effectively elucidates the observed antioxidant and anti-inflammatory properties of ADNVs in studies involving human cell lines.

### Farming practices influence ADNVs composition and cargo

Organic farming has been proposed, in this article, as a variable for ADNVs characteristics in opposition to canonical farming practices. Organic farming affect mycorrhizal relationships and subsequently fruit development and maturation. Quantification analysis of GD and GDBio-derived ADNVs reports how the farming practice determines not ADNVs’ size distribution or subpopulation composition, but their yield. Apples from organic farming produced 100 times more EVs than the ones derived from integrated farming (Fig. 1B). Considering that PDEVs are part of mycorrhizal exchanges [10, 11] and that organic farming enhances the mycorrhizal network, higher production of ADNVs under organic farming could be explained by the need for larger traffic of information.

The identified miRNA families are the same in GD and GDBio samples: the distinction lies, again, in the relative abundance of each species (Additional file 1: Figures S2A and S2B). miRNA's expression can be linked to several aspects of fruit development, including ripeness and conservation. For this reason, changes in miRNA levels are expected. For instance, mdm-miR482 and mdm-miR396, which we found in ADNVs, are most expressed in mature and young fruits respectively [59].

Lipid composition differs slightly in the two conditions. GDBio contains more lysoglycerophospholipids (LPs), specifically LPC and LPE (Additional file 1: Figure S3B), that well-known bioactive lipids. LPs act as extracellular mediators, thus mediating various cellular physiological responses [65]. In pharmacology, they are extensively used for their capability to promote wound healing in vitro and their compatibility with mammalian cells [66, 67]. Their presence in ADNVs could be crucial for the interaction with recipient cells in all eukaryotes. Further studies should consider investigating whether the lipidic content of GD and GDBio influences their effect. Given that ADNVs can have multiple health and agriculture-related uses, further studies should consider investigating whether the lipidic content of GD and GDBio influences their effect and, thus, their applications.

## Conclusions

In conclusion, the ADNVs fraction is mainly composed of exocyst-positive organelles (EXPOs) and MVB-derived exosomes, identified through size and molecular markers (Exo70 and TET-3-like proteins). Observing their cargo, it is possible to say that ADEVs contribute to multiple physiological aspects of the plant: (i) they present a wide variety of defence‐related cargoes, playing key roles in plant–pathogen interactions and in abiotic stress resistance, (ii) they are involved in cell communication, (iii) cell wall remodelling and (iv) in controlling ROS levels. When interacting with human cell lines, such as fibroblasts, ADNVs trigger a wave of intracellular Ca^2+^ signalling, deriving from intracellular compartments. Calcium signalling is couples with the ADNVs antioxidant activity, observed with DPPH assay and by monitoring mitochondrial superoxide levels. Enzymes carried by ADNVs, such as peroxidase, might have a role in the perpetuation of ADNVs biological effect on human cell lines. Lastly, the influence of cultivation practices on ADEVs development is significant in terms of yield, higher in GDBio.

### Supplementary Information

**Additional file 1: Supplementary Figures. **

**Additional file 2: Supplementary Tables. **

## Data Availability

Data and materials are available following requirement.
