# Supplementary material for: Link between organic nanovescicles from vegetable kingdom and human cell physiology: intracellular calcium signalling
Source: J Nanobiotechnology. 2024 Feb 19;22:68. doi: 10.1186/s12951-024-02340-8 (PMC10875884; doi:10.1186/s12951-024-02340-8)
Supplement: Supplementary file 1 — Additional file 1: Supplementary Figures. [file 12951_2024_2340_MOESM1_ESM.pptx]

## Slide 1
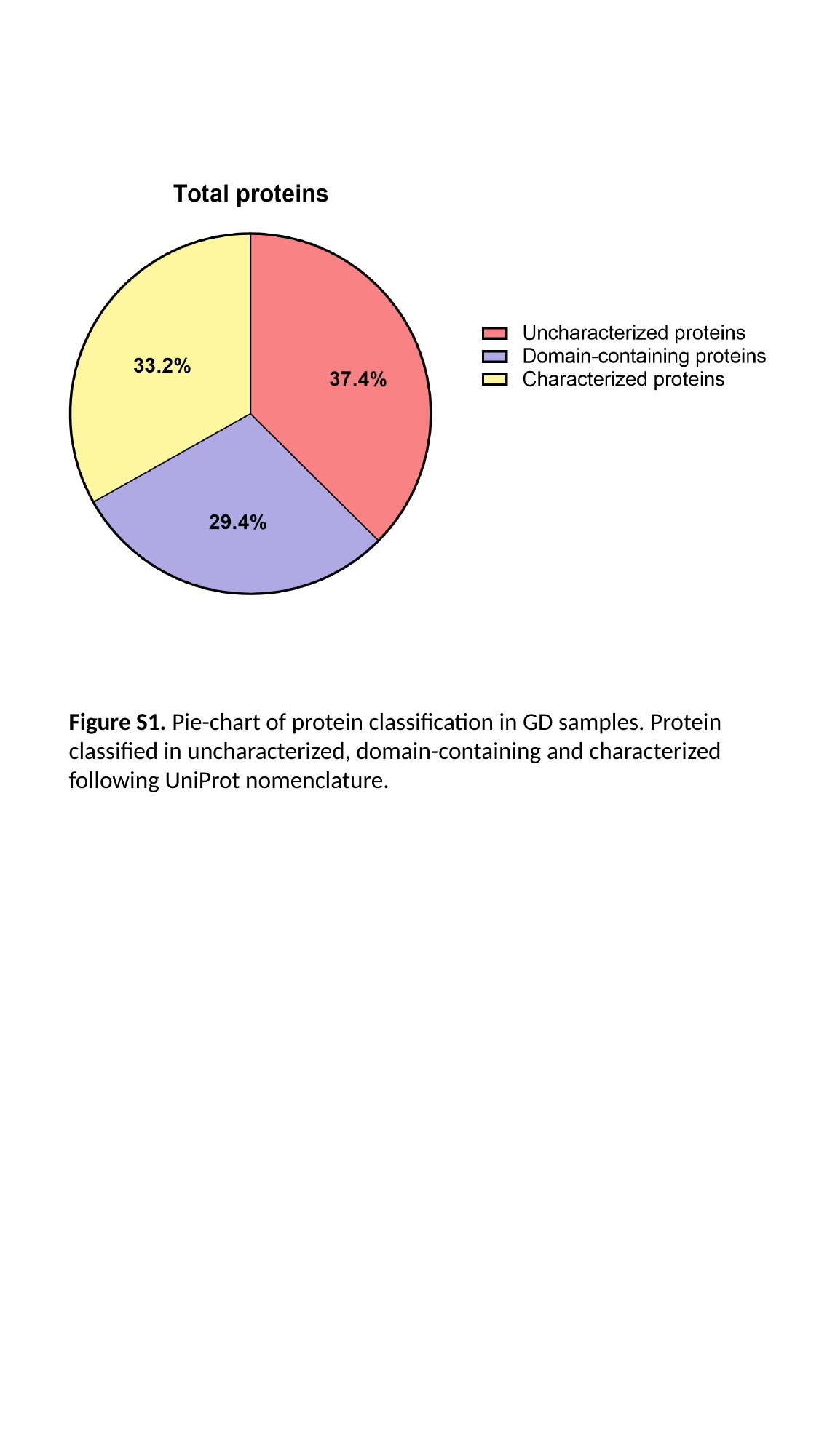

Figure S1. Pie-chart of protein classification in GD samples. Protein classified in uncharacterized, domain-containing and characterized following UniProt nomenclature.

## Slide 2
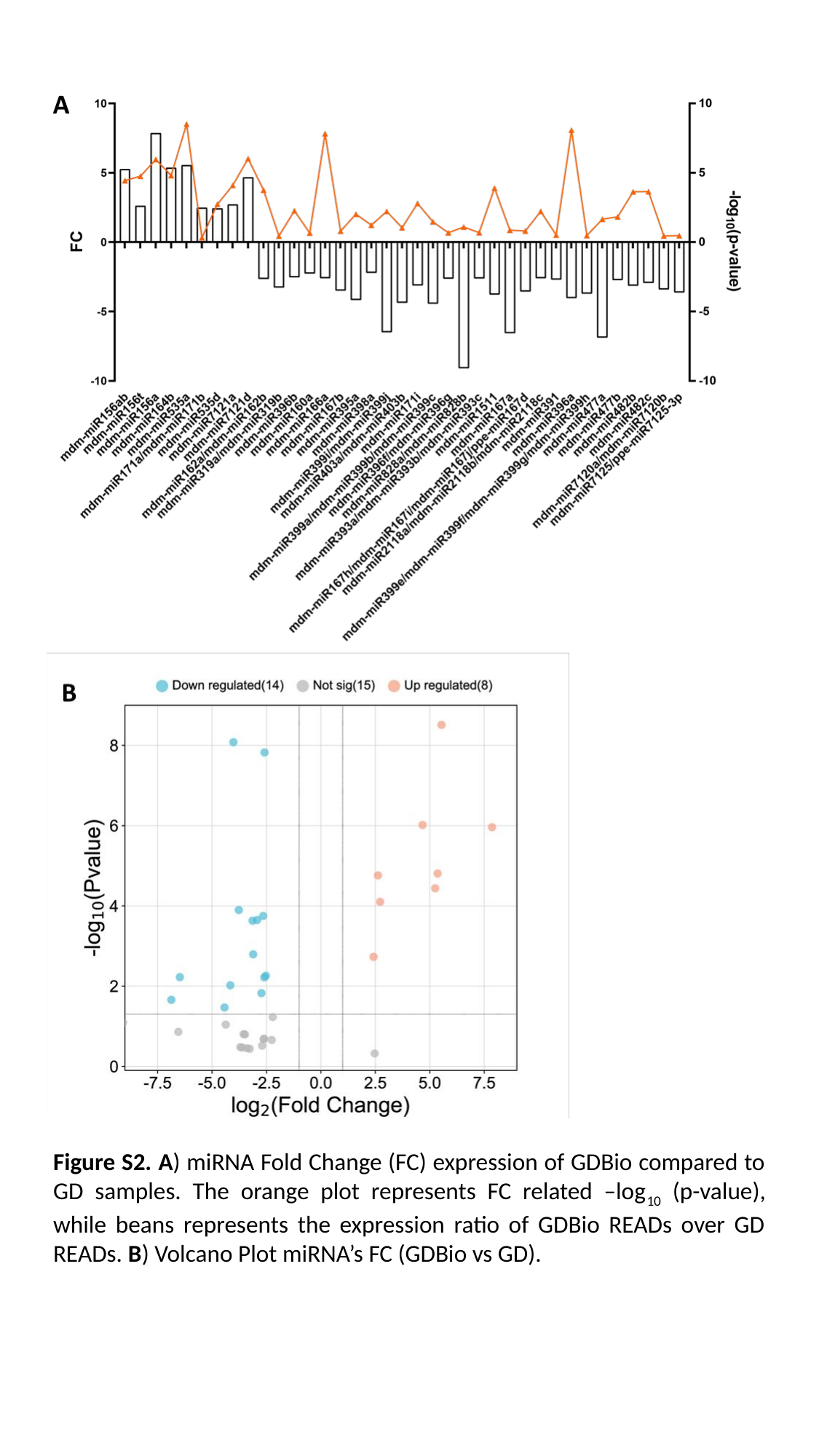

Figure S2. A) miRNA Fold Change (FC) expression of GDBio compared to GD samples. The orange plot represents FC related –log10 (p-value), while beans represents the expression ratio of GDBio READs over GD READs. B) Volcano Plot miRNA’s FC (GDBio vs GD).

## Slide 3
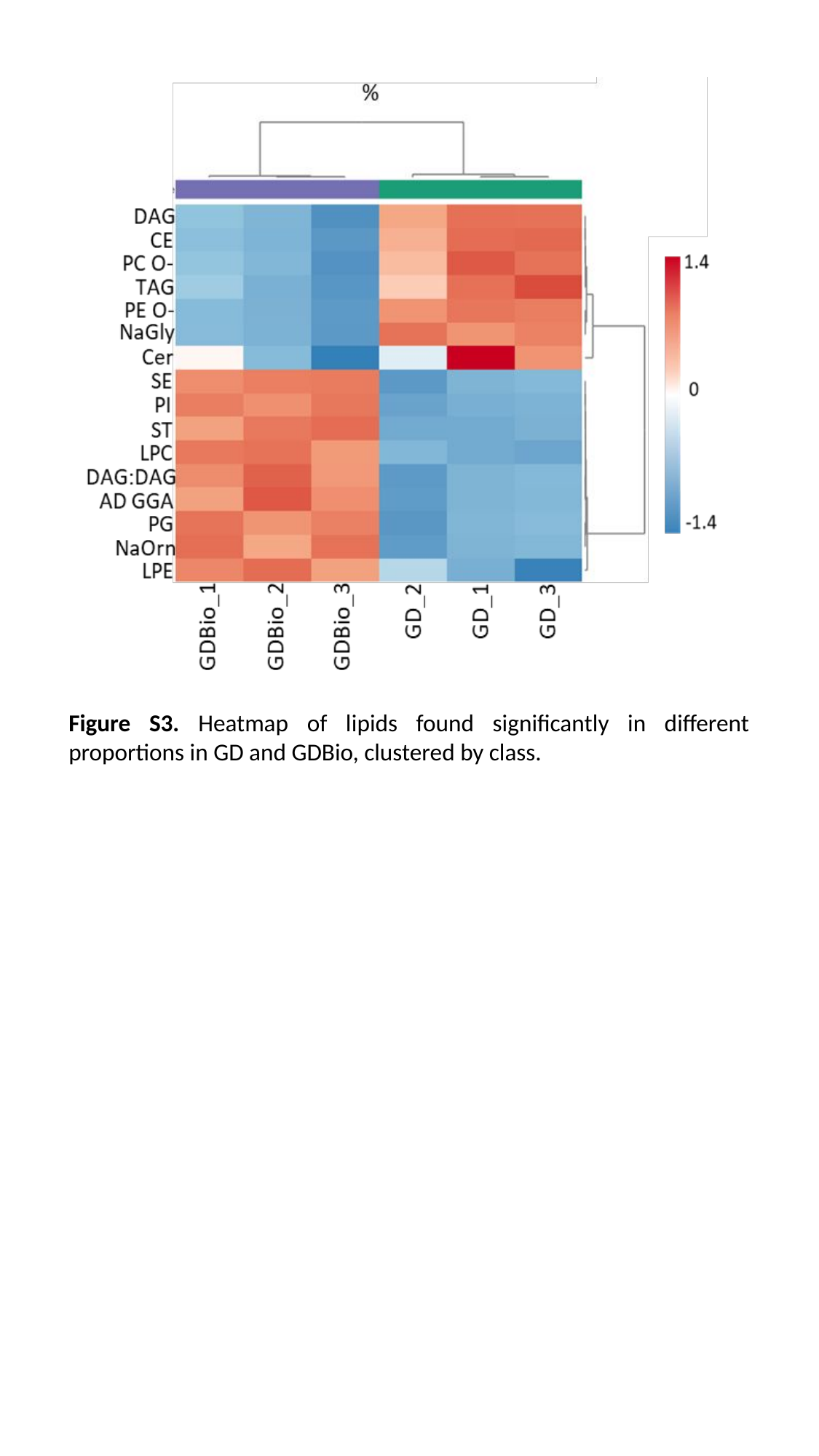

Figure S3. Heatmap of lipids found significantly in different proportions in GD and GDBio, clustered by class.

## Slide 4
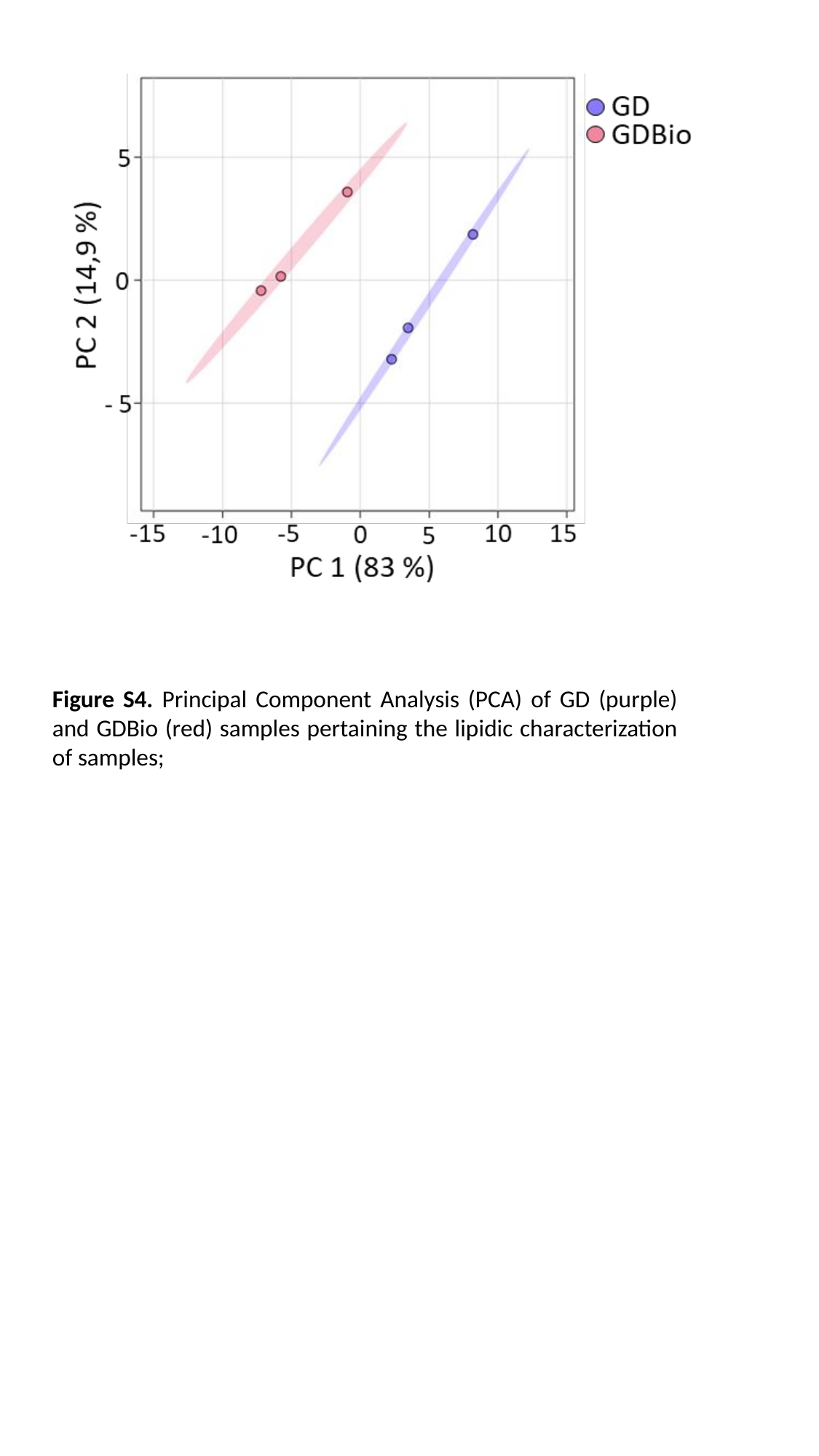

Figure S4. Principal Component Analysis (PCA) of GD (purple) and GDBio (red) samples pertaining the lipidic characterization of samples;

## Slide 5
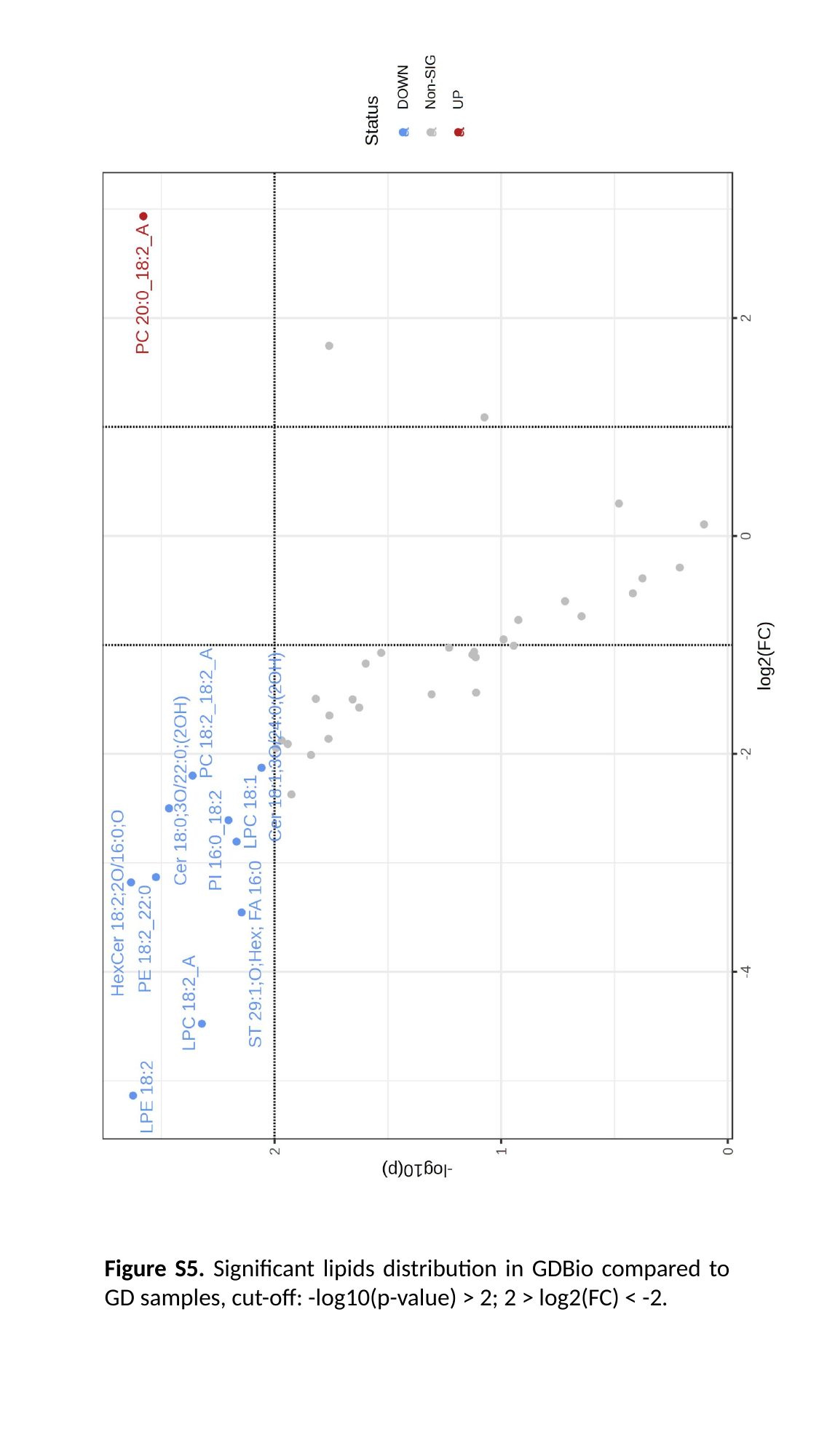

Figure S5. Significant lipids distribution in GDBio compared to GD samples, cut-off: -log10(p-value) > 2; 2 > log2(FC) < -2.

## Slide 6
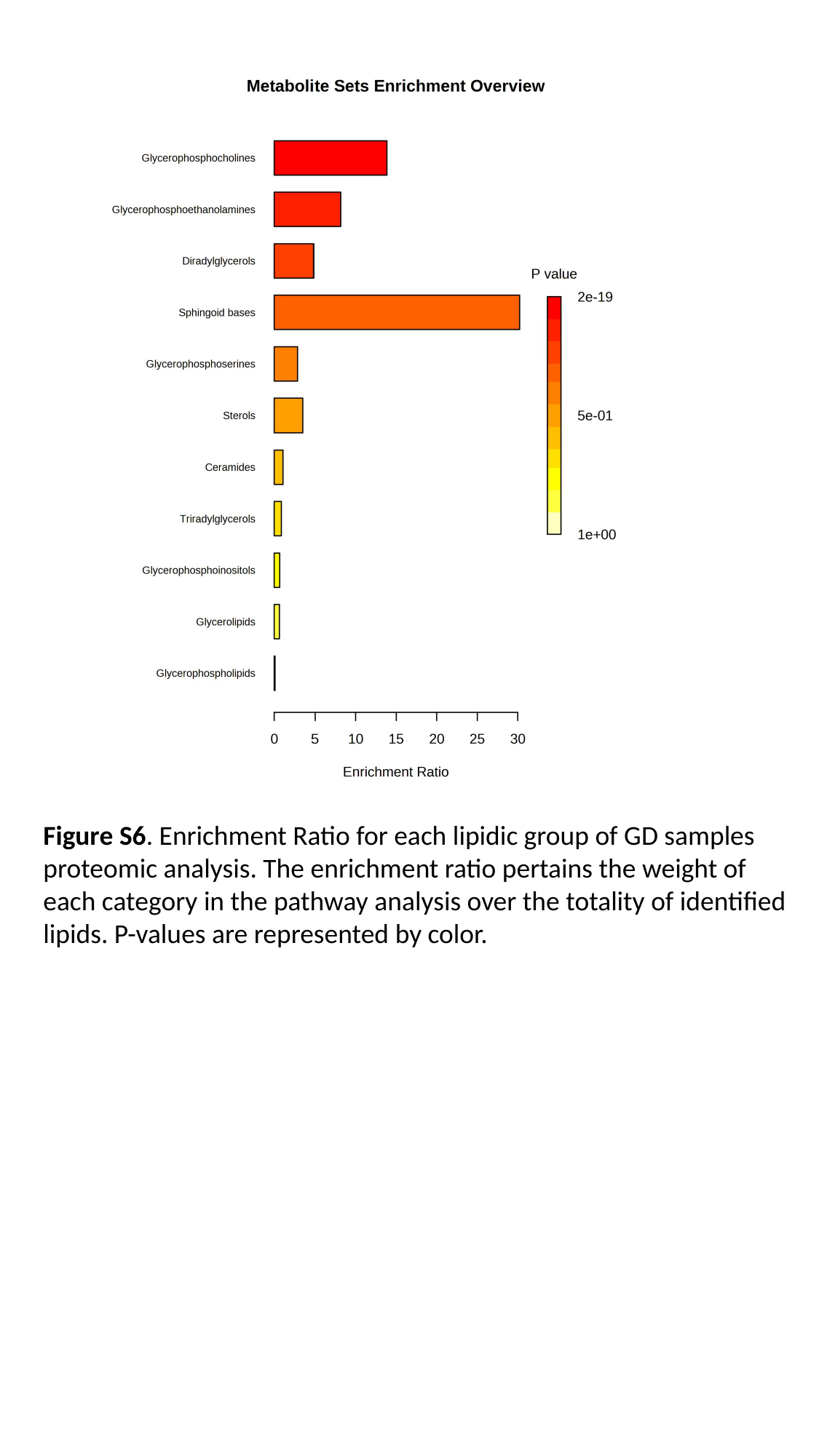

Figure S6. Enrichment Ratio for each lipidic group of GD samples proteomic analysis. The enrichment ratio pertains the weight of each category in the pathway analysis over the totality of identified lipids. P-values are represented by color.
